# Adolescents' trajectories of mental health in the MYRIAD trial

**DOI:** 10.1002/jcv2.70046

**Published:** 2025-10-07

**Authors:** Carolina Guzman Holst, Simona Skripkauskaite, Jack L. Andrews, Jesus Montero‐Marin, Verena Hinze, Tim Dalgleish, Willem Kuyken, MYRIAD team, Lucy Foulkes

**Affiliations:** ^1^ Department of Experimental Psychology University of Oxford Oxford UK; ^2^ Department of Psychiatry University of Oxford Oxford UK; ^3^ Teaching Research & Innovation Unit Parc Sanitari Sant Joan de Déu Sant Boi de Llobregat Spain; ^4^ Consortium for Biomedical Research in Epidemiology & Public Health (CIBER Epidemiology and Public Health ‐ CIBERESP) Madrid Spain; ^5^ Medical Research Council Cognition and Brain Sciences Unit University of Cambridge Cambridge UK

**Keywords:** adolescence, depression, mindfulness training, school‐based interventions, wellbeing

## Abstract

**Background:**

This study explored adolescent's mental health trajectories over the course of a school‐based mindfulness‐based intervention trial (MYRIAD). It examined whether intervention condition (mindfulness vs. teaching‐as‐usual), individual‐level and contextual‐level factors were associated with different trajectories.

**Methods:**

This pre‐registered study used data from 11‐ to 14‐year‐olds who participated in the MYRIAD trial. We used growth mixture models to examine distinct trajectories in risk for depression, social‐emotional‐behavioural functioning, and wellbeing (co‐primary outcomes), and anxiety (secondary outcome), across pre‐intervention, post‐intervention and 12‐month follow up (ns = 7198–7727). We then used multinomial and binomial logistic regression models to examine factors associated with individual trajectory membership.

**Results:**

Distinct trajectories emerged for each outcome: A five‐trajectory model best explained the changes in risk for depression, whilst four‐trajectory models best explained changes in social‐emotional‐behavioural functioning, wellbeing, and anxiety. While 69%–80% of adolescents followed stable low‐problem trajectories for each outcome, 11%–23% experienced stable high‐problem trajectories, 2%–16% experienced increasing‐problem trajectories and 1%–5% experienced decreasing‐problem trajectories. Receiving the mindfulness intervention was not associated with any mental health trajectory in models adjusted for confounders. Several individual‐level factors, including executive functioning difficulties and risk of mental health problems at baseline, and school‐level factors, such as school climate, predicted adolescents' classification into different trajectories, but they did not vary according to intervention group.

**Conclusions:**

Individual differences in mental health trajectories emerged over the course of a 1‐year mindfulness‐based intervention, with most adolescents experiencing low‐stable problem trajectories for each outcome. However, the intervention itself had no impact on individual trajectory membership, mirroring null results found in the main trial. Our findings suggest that universal interventions may not be sensitive enough to address the diverse needs of all students, however, tailoring interventions to address a range of different individual and contextual factors might maximise their impact.

## Key Points

**What's known?**

- The MYRIAD trial found no overall mental health benefit of school‐based mindfulness training versus teaching as usual. However, average results may be masking individual differences in how young people responded to the intervention over time.

**What's new?**

- This study used a data‐driven approach to investigate adolescents' mental health and wellbeing trajectories over the course of a 1‐year, school‐based mindfulness intervention (MYRIAD), and factors associated with trajectory membership. This is an essential step in better understanding how an intervention works and for whom.

**What's relevant?**

- In a large, broadly representative sample of adolescents in the UK, we found significant variability in mental health trajectories, with most individuals following trajectories of low‐stable mental health problems and average wellbeing. Different levels or types of interventions might be needed to adequately address the wide range of adolescents' mental health needs.
- Targeting factors such as school climate (e.g. school safety and teachers' engagement in procuring a positive environment) prior to an intervention, or modifying the intervention to be suitable for individuals with different pre‐existing levels of mental health problems or executive functioning difficulties might improve their intervention outcomes and mental health trajectories.
- Trajectory models provide unique insight into the prevalence and progression of mental health problems across an intervention sample, and thus should become a standard practice when examining short‐term and long‐term intervention effects.

## INTRODUCTION

Many school‐based mental health programmes have been developed in the past decade to improve mental health in adolescence. These include universal interventions, which are delivered to all school pupils regardless of need (Greenberg & Abenavoli, 2017). Universal mental health interventions typically incorporate psychoeducation and practical coping strategies and are aimed at preventing mental health problems from emerging or reoccurring, reducing symptoms for those already experiencing problems, and/or promoting wellbeing (Salazar de Pablo et al., 2020; Vostanis et al., 2013).

Evidence indicates that on average school‐based universal interventions can improve young people's mental health, although effect sizes are generally small or inconsistent (Werner‐Seidler et al., 2021; Zhang et al., 2023). For example, a systematic review and meta‐analysis of 43 randomised controlled trials of mindfulness‐based universal interventions found small effects on anxiety and stress immediately post‐intervention (*d* = 0.11), but no significant effects on depression and wellbeing, and no significant effects of mindfulness programmes at the follow‐up timepoint between one and 40 months later (Dunning et al., 2022). Notably, one of the largest mindfulness‐based school trials, the My Resilience in Adolescence (MYRIAD) study, found no differences in primary mental health outcomes between students in the mindfulness intervention (SMBT) and the usual social‐emotional teaching (teaching as usual; TAU) control group, either immediately post‐intervention or at the 1‐year follow‐up (Kuyken et al., 2022). These findings have prompted some researchers to consider that universal school‐based mental health interventions may not be an appropriate way to reduce mental health problems in young people (Cuijpers, 2022).

However, most studies evaluating school‐based mental health interventions have not considered individual differences. It is possible that while some young people are benefitting from these interventions, others are not, or may even experience negative effects (Foulkes & Stringaris, 2023; Guzman Holst et al., 2024) — and such variation may be masked when only average effects are reported. Adolescents can be heterogeneous in both their initial levels of risk and their developmental trajectories over time. Ignoring this variability can underestimate the potential impact of universal interventions and overlook subgroups for whom the intervention may be particularly beneficial or, conversely, potentially harmful (Greenberg & Abenavoli, 2017). Therefore, examining intervention effects using methods such as latent trajectories (i.e., distinct and empirically‐derived time courses) of mental health symptoms can allow us to better understand potential individual differences in response to an intervention over time. While other methods such as subgroup analyses are indeed well established in school‐based intervention research and may offer information on how main effects are moderated, they do not examine individual‐level heterogeneity in trajectories over time. Subgroup analyses are variable‐centred, meaning they can only describe how factors relate over time and assume that all individuals with these factors respond the same way. Instead, person‐centred approaches such as LGMM may explain how individuals rather than factors individually respond over time (Howard & Hoffman, 2018). Thus, relying solely on point estimates or subgroup analyses may not adequately capture for whom interventions are effective or ineffective, limiting our understanding of differential intervention effects.

A growing body of research has examined developmental trajectories of adolescent mental health and wellbeing, identifying both stable and changing patterns across this critical period. For example, a meta‐analysis examining individual differences in the development of depressive symptoms found that individual studies identified anywhere between 3 and 11 trajectories (Shore et al., 2018). However, more often studies typically report 4‐6 trajectories which are characterised by low levels of symptoms sustained over time, moderate levels sustained over time, increasing or decreasing levels across time and high levels of mental health difficulties (Korhonen et al., 2014; Musliner et al., 2016; Patalay et al., 2015; Schubert et al., 2017; Shore et al., 2018). Notably, this demonstrates that children may fluctuate in their risk for mental health‐related problems over time. In the present study our objective is to extend the application of these trajectory analyses to a school‐based intervention context, which remains underexplored in the existing literature.

There is also a need to understand what risk and protective factors predict specific trajectories of mental health outcomes. For example, individual‐level predictors such as having a pre‐existing mental health problem might increase the risk of following trajectories of symptom decline over the course of an intervention (Montero‐Marin et al., 2022; Stallard et al., 2013). Executive function may also be important: some evidence suggests that higher levels of executive function might mediate the relationship between mindfulness training and mental health by increasing cognitive flexibility and emotion regulation (Chiesa et al., 2011; Hölzel, Carmody, et al., 2011). It is also theorised that mindfulness practices may be more effective at reducing mental health problems and increasing wellbeing for those who are already able to focus and sustain attention on breathing or bodily feelings for a longer period, or who are more adept at regulating their emotions (Hölzel, Lazar, et al., 2011).

Other individual‐level predictors such as gender, ethnicity and school‐level predictors such as school climate and socio‐economic deprivation (e.g. percentage of free school meals in a school), may also determine trajectories of symptom decline or improvement. Existing evidence indicates that girls may respond better and benefit more from mindfulness training than boys (Kang et al., 2018; Montero‐Marin et al., 2023), thus increasing their likelihood of being in a trajectory group of symptom improvement. Likewise, some studies (DeLuca et al., 2018; Sun et al., 2022) suggest that mindfulness‐based interventions could have differential effects on different ethnic groups, with some minority groups potentially benefitting more; however, this area has been largely unexplored.

Students' perceptions of school climate may also impact the response to an intervention. There is some qualitative evidence that young people who feel unsafe in the classroom may be less likely to engage in mindfulness practices at school (Hailwood, 2020), suggesting that those who have negative perceptions of their school climates may be less likely to experience symptom improvements in mindfulness‐based interventions. Equally, those who perceive their school climate positively might be more likely to experience symptom improvements, because the supportive environment is more conducive to learning mindfulness skills with peers. Regarding deprivation, in the MYRIAD study, higher school‐level economic deprivation was related to greater intervention responsiveness (i.e. positive attitudes and interest towards SBMT), potentially indicating that mindfulness might be most impactful for schools with fewer resources (Montero‐Marin et al., 2023). Lastly, broader school‐level contextual factors such as urbanicity (whether a school is in a rural or urban area) have also been associated with mental health outcomes (Ford et al., 2021; Hinze et al., 2024). In the MYRIAD study, students in schools in rural areas had lower socio‐emotional‐behavioural difficulties and higher wellbeing at baseline than those in urban areas, and this association remained stable over time (Hinze et al., 2024). It is likely that young people living in urban areas are exposed to more social stressors such as inequality, crime and familial isolation, which may impact mental health (Ford et al., 2021; Okkels et al., 2018).

Therefore, this study sought to understand how different individual‐level, school‐level and broader contextual factors predicted young people's symptom trajectories during the course of a 1‐year trial assessing a mindfulness intervention.

### Objectives

Our three research questions were:

1. Are there multiple distinct trajectories in young people's risk for depression, social‐emotional‐behavioural functioning, wellbeing (primary outcomes) and anxiety (secondary outcome) over the course of a school‐based mindfulness trial, from pre‐to post‐intervention to 1‐year follow up?
2. Does receiving a school‐based mindfulness training intervention (SBMT) or teaching as usual (TAU) predict young people's classification into different trajectories of risk for depression, social‐emotional‐behavioural functioning, wellbeing or anxiety?
3. Can school‐level or individual‐level factors predict young people's classification into different trajectories, and do they vary according to intervention group?

## METHODS

### Design

We conducted a secondary analysis using data from the MYRIAD trial, a two‐arm parallel group cluster randomised controlled trial (ISRCTN86619085; 03/06/2016). The trial began in 2016 and recruitment took place in two cohorts in the academic years of 2016/2017 and 2017/2018. A total of 84 secondary schools and 8376 adolescents (age 11–13 years at baseline) were recruited across the UK. Schools were randomised into one of two conditions: universal school‐based mindfulness training (SBMT; *N* = 4232 in 43 schools) and socio‐emotional teaching as usual (TAU, *N* = 4144 in 42 schools). Assessments were taken just before randomisation (baseline), immediately prior to the intervention (pre‐intervention) and 12 months after pre‐intervention (12‐month follow up). All of our outcome measures were selected to mirror the main trial and were assessed at three time points: pre‐intervention, post‐intervention and 12‐month follow up (see Supporting Information S1: Figure S1). Individual and school‐level predictors were assessed at either baseline or pre‐intervention. The SBMT consisted of 10 sessions delivered during the spring term (January–April). Details on the eligibility, recruitment, procedure, intervention conditions, teacher training and participants can be found in the study protocol and protocol update (Kuyken et al., 2017; Montero‐Marin et al., 2021).

### Participants

The current study focuses on a sub‐sample of young people who completed an assessment at pre‐intervention and at least one assessment at either post‐intervention or follow‐up; this was done to reduce potential biases related to attrition, as detailed in the pre‐registration (Guzman Holst et al., 2023). Details on sample size and sample characteristics can be found in Table 1. Details on attrition can be found in the main study (Kuyken et al., 2022). Details on missing data and data availability can be found in Supporting Information S1: Table S1.

**TABLE 1:** Baseline/pre‐intervention characteristics of students by trial arm status and overall.

Student characteristics	SBMT (*n* = 4232)	TAU (*n* = 4144)	Total (*n* = 8376)
Gender			
Female, *n* (%)	2350 (56.5)	2159 (53.1)	4509 (54.9)
Male, *n* (%)	1724 (41.5)	1823 (44.9)	3547 (43.2)
Other, *n* (%)	14 (<1.0)	12 (<1.0)	26 (<1.0)
Prefer not to say	69 (1.7)	69 (1.7)	138 (1.7)
Age, mean (sd)	11.7 (0.6)	11.7 (0.6)	11.7 (0.6)
Ethnicity			
Arab/Arab British, *n* (%)	80 (1.9)	80 (2.0)	160 (2.0)
Asian/British Asian, *n* (%)	357 (8.6)	482 (11.9)	839 (10.2)
Black/Caribbean/Black British, *n* (%)	191 (4.6)	228 (5.6)	419 (5.1)
Mixed/multiple ethnic groups, *n* (%)	183 (4.4)	191 (4.7)	374 (4.5)
White, *n* (%)	3237 (78.1)	2965 (73.2)	6202 (75.7)
Other ethnic group, *n* (%)	97 (2.3)	102 (2.5)	199 (2.4)
Executive functioning (BRIEF), mean (SD)	84.0 (21.1)	83.3 (20.4)	83.7 (20.8)
Risk of mental health			
Low risk, *n* (%)	3056 (72.2)	3045 (73.7)	6101 (72.8)
High risk, *n* (%)	1176 (27.8)	1099 (26.5)	2275 (27.2)
Depression (CESD‐D), mean (SD)	15.6 (11.2)	15.6 (10.9)	15.6 (11.1)
Social‐emotional and behavioural functioning (SDQ)	12.5 (6.7)	12.3 (6.5)	12.4 (6.6)
Wellbeing (WEMWBS), mean (SD)	49.2 (9.2)	49.0 (9.0)	49.1 (9.1)
Anxiety (RCADS), mean (SD)	28.7 (20.2)	27.9 (19.7)	28.3 (20.0)

School characteristics	SBMT (*n* = 41)	TAU (*n* = 43)	Total (*n* = 84)
Deprivation status (% eFSM), mean (SD)	13.2 (8.1)	11.8 (10.7)	12.5 (9.4)
Urbanicity			
Urban, *n* (%)	35 (85.4)	36 (85.4)	71 (84.5)
Rural, *n* (%)	6 (14.6)	7 (16.3)	13 (15.5)
School climate (SCCS), mean (SD)	3.38 (0.6)	3.36 (0.6)	3.37 (0.6)

*Note*: In the intervention arm 4157 students provided data on gender, 4145 students provided data on ethnicity, 4230 students provided data on CES‐D, 4171 on SDQ and 4214 on WEMWBS. In the control arm, 4063 students provided data on gender, 4048 students provided data on ethnicity, 4140 students provided data on CES‐D, 4081 on SDQ and 4119 on WEMWBS. School climate is student‐rated will be aggregated at the school level. The mean is individual level. WEMBS, Depression, SDQ and RCADS means at pre‐intervention. The subsample used in this study is broadly representative of adolescents in the UK. Indicators such as gender, ethnicity and %eFSM are broadly in line with national statistics (see Department of Education, Schools and Pupil Statistics Reports).

Abbreviations: BRIEF, Behaviour Rating Inventory of Executive Function; CES‐D, Centre for Epidemiologic Studies Depression Scale; eFSM, eligibility for free school meals; RCADS, Revised Children's Anxiety and Depression Scale; SBMT, School‐based mindfulness training; SCCS, School Climate and Culture Survey; SD, standard deviation; SDQ, Strengths and Difficulties Questionnaire; TAU, teaching as usual; WEMWBS, Warwick‐Edinburgh Mental Wellbeing Scale.

### Measures

Details regarding the cut‐off scores of each measure are given below. Full details of all the measures are presented in Supporting Information S1.

#### Primary and secondary outcome measures

##### Depressive symptoms

Participants completed the Centre for Epidemiological Studies for Depression Scale (CES‐D) (Radloff, 1991). The CES‐D provides cut‐off scores for low risk of depression (0–15), at risk of depression (16–27) and probable caseness (28–60) (Radloff, 1991). To maintain consistency with other papers in the MYRIAD trial we refer to this as ‘risk for depression.’

##### Wellbeing

Participants completed the Warwick‐Edinburgh Mental Wellbeing Scale (WEMWBS) (Tennant et al., 2007). The WEMWBS provides cut‐off scores for probable mental health difficulties (0–40), possible mental health difficulties (41–44), average wellbeing (45–59) and high wellbeing (60–70) (Tennant et al., 2007).

##### Social‐emotional‐behavioural difficulties

Participants completed the self‐reported youth version of the Strengths and Difficulties Questionnaire (SDQ) (Goodman, 1997, 2001). Total scores were interpreted based on the new 4‐categorisation band for the self‐reported SDQ: low (0–14), moderate (15–17), high (18–19) and very high (20–40) (Youth in Mind).

##### Anxiety

Participants completed the anxiety subscales from the Revised Child Anxiety and Depression Scale (RCADS) (Chorpi et al., 2000). Raw scores were analysed continuously. We use the descriptors ‘high’, ‘moderate’ and ‘low’ to describe and visualise trajectories, however this should be interpreted with caution given that raw scores were used for the RCADS rather than t‐scores; these labels do not necessarily equate quantitatively to clinical severity.

#### Individual‐level, school‐level and contextual predictors

Individual‐level predictors included demographic information (age, gender and ethnicity), executive functioning (BRIEF‐2) (Gioia et al., 2000), and risk of mental health problems (variable derived from latent profile analysis) (Howard & Hoffman, 2018). School‐level and contextual predictors included school climate (SCCS) (School Climate and Connectedness Survey) derived by aggregating student ratings, School‐level deprivation (percentage of students eligible for free school meals; eFSM) (Kuyken et al., 2022), and urbanicity (rural vs. urban government classification) (Kuyken et al., 2022).

### Statistical analysis

#### Latent trajectory analysis

For our main analysis we used latent trajectory modelling in Mplus version 8.10 (Muthén, 2023) to identify potentially different trajectories of mental health outcomes throughout the course of a school‐based mindfulness trial. Four outcomes—risk for depression, social‐emotional‐behavioural functioning, wellbeing and anxiety—were modelled separately to retain specificity, avoid multicollinearity in join modelling and reduce common method bias. A time variable was devised to track intervention progression, which was equally spaced between 6‐month intervals (at a maximum), including pre‐intervention (Time 0), post‐intervention (Time 1) and follow‐up (Time 2). Models were estimated using full information maximum likelihood estimation (FIML), which takes into account any of the missingness in the data without discarding any information to produce unbiased estimates. FIML is usually recommended over multiple imputation (MI) methods for latent trajectory modelling as it facilitates model convergence and outperforms other methods such as MI when missingness levels are high (Enders, 2010; Xiao & Bulut, 2020).

We first modelled individual data and separate latent growth curves for each outcome to determine whether linear or quadratic polynomial functions best fit the data. We then fit a series of models beginning with one single growth curve trajectory (our overall mean trajectory) and successively added latent classes until we reached an optimal number of groups. We set an upper limit of 6 classes, as mental health trajectories estimated across different populations typically identify between 4‐6 groups (Korhonen et al., 2014; Musliner et al., 2016; Schubert et al., 2017; Shore et al., 2018). Following this approach, we compared the best fitting model using latent class growth analysis (LCGA) with all within‐class variances for intercepts and slopes fixed to 0, to a constrained general growth mixture model (GGMM) with within‐class variances in which the intercepts are equal but freely estimated and the slopes are fixed to 0 (Supporting Information S1: Table S2–S5). The best fitting models were a mixture of four‐class linear GGMM (for social‐emotional‐behavioural functioning and wellbeing), a five‐class GGMM (for risk for depression) and a four‐class quadratic GGMM (for anxiety) based on the statistical indices described below (see Table 2). These models were then used for all further analysis.

**TABLE 2:** Indices for selected models and class sizes.

Model	Class	AIC	BIC	Entropy index	VLMR LRT
CESD, GMM ‐ Q	5	157,094.29	157,254.16	0.82	410.24, *p* < 0.001
SDQ, GMM ‐ L	4	133,320.84	133,425.07	0.66	255.36, *p* < 0.001
WEMWBS, GMM ‐ L	4	153,205.82	153,310.11	0.68	124.84, *p* = 0.013
RCADS, GMM ‐ Q	4	168,886.66	169,017.41	0.83	550.89, *p* < 0.001

Model	Class	N (%)	Description
CES‐D	1	394 (5.1)	Low to risk to caseness, increasing symptoms
	2	606 (7.9)	Risk to caseness to risk, increase then decrease in symptoms
	3	400 (5.2)	Caseness to risk to risk, decreasing then stable symptoms
	4	931 (12.1)	Caseness to caseness to caseness, high stable symptoms
	5	5381 (69.8)	Low to low to low, low stable symptoms
SDQ	1	700 (9.1)	Low to average to very‐high, increasing difficulties
	2	4938 (64.2)	Low to low to low, stable difficulties
	3	325 (4.2)	Very‐high to average to low, decreasing difficulties
	4	1734 (22.5)	High to very‐high to very‐high, stable difficulties
WEMWBS	1	1210 (15.7)	Possible to possible to probable, decrease in wellbeing
	2	200 (2.6)	Average to possible to probable, decrease in wellbeing
	3	139 (1.8)	Probable to average to average, increase in wellbeing
	4	6175 (79.9)	Average to average to average, stable wellbeing
RCADS	1	5514 (76.6)	Low to low to low, low stable anxiety
	2	541 (7.5)	Moderate to high to high, increase in anxiety
	3	354 (4.9)	High to moderate to moderate, decrease in anxiety
	4	789 (11.0)	High to high to high, high stable anxiety

Abbreviations: AIC, Aikaike Index Criteria; BIC, Bayesian Information Criterion; CES‐D, Centre for Epidemiologic Studies Depression Scale; GMM, growth mixture model; L, linear; Q, quadratic; RCADS, Revised Children's Anxiety and Depression Scale; SDQ, Strengths and Difficulties Questionnaire; VLMR LRT, Vuong‐Lo‐Mendell‐Rubin Likelihood Ratio Test; WEMWBS, Warwick‐Edinburgh Mental Wellbeing Scale.

To determine the best statistical fit with models that had the same number of classes we used the likelihood‐ratio chi‐square difference tests with Satorra‐Bentler correction (Satorra & Bentler, 2010). To compare models with a different number of classes and determine the optimal number of groups we used the Bayesian Information Criterion (BIC), the Vuong‐Lo‐Mendell‐Rubin Likelihood Ratio Test (VLMR‐LRT) (Lo et al., 2001), and the Entropy Index. Specifically, we used a lower BIC value, a significant VLMR‐LRT *p*‐value comparing *k* (the number of classes determined by the model), and k‐1 class (the previous model with one less class), and a higher entropy value (near 1.0), in that order to guide our decision. Other considerations included successful convergence, class size (with a pre‐specified minimum size of 100 individuals per class), theoretical grounds and ease of interpretability. All our models converged using 1000 random sets 100 optimisations and 100 start iterations.

### Multinomial/binomial logistic regression analysis

To answer our second and third research questions regarding predictors, we regressed trajectory group membership, obtained from the individual posterior probability classification, onto different baseline individual‐level and school‐level variables. We first used multinomial logistic regressions for each outcome using the largest trajectory group and then used binomial logistic regression to examine direct comparisons between all increasing and decreasing problem trajectory groups, as specified in our pre‐registration (Guzman Holst et al., 2023). We did not use hierarchical models, in spite of the clustered nature of the data, since random effects models failed to converge due to singularity (i.e. the model was overfitted). Intraclass correlation coefficients confirmed that only a very small amount of variance in each outcome score was due to school‐level effects (ICC range: 0.007‐0.018), indicating that it is less likely to produce any type‐1 error or conflation effects in our coefficients (Musca et al., 2011). To examine the impact of the intervention condition we present both unadjusted and adjusted models. Unadjusted models only included the intervention condition whilst adjusted models included intervention condition, individual‐level variables (gender, age, ethnicity, executive function, risk of mental health problems) and school‐level variables (school climate, school‐level deprivation, urbanicity). We also included separate interactions between intervention condition and each predictor variable. Class probability weights were included in all regression models to account for any reduced accuracy in classification. We a priori set the alpha level to *α* < 0.05 and adjusted *p*‐values for multiple comparisons using the False Discovery Rate (FDR) correction (Benjamini & Hochberg, 1995) in all analyses. These analyses were performed in R (Team, 2018).

## RESULTS

### Trajectories of risk for depression‐behavioural functioning, wellbeing and anxiety

**FIGURE 1:**
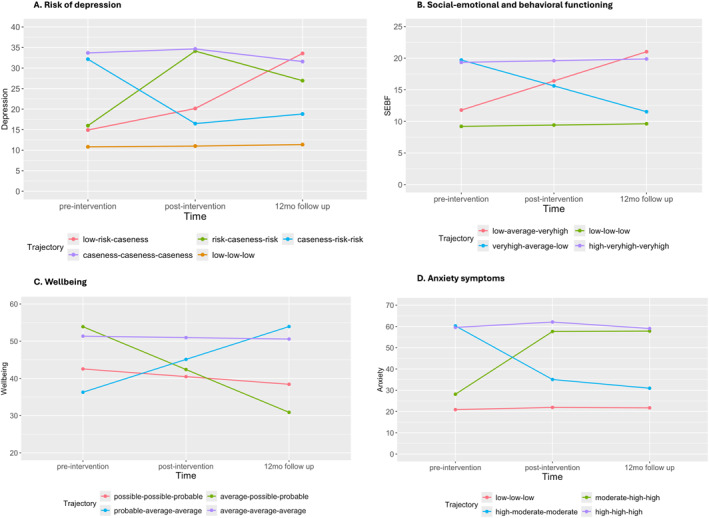
Trajectories of all co‐primary and secondary outcomes across a 1‐year mindfulness intervention. For details regarding class sizes see Table 2. Trajectory labels for anxiety symptoms do not necessarily equate to quantitative clinical severity. Sample size (N) varied for each outcome; risk of depression = 7712; socio‐emotional‐behavioural functioning = 7697, wellbeing = 7724, anxiety symptoms = 7198.

Model indices and class descriptions for all outcomes can be found in Table 2. For risk for depression, the best fitting model was a five‐trajectory model (Figure 1A), labelled according to the CES‐D scale thresholds (Radloff, 1991). This model identified a low‐stable symptom group (‘low‐low‐low’, *n* = 5381), a high‐stable symptom group (‘case‐case‐case’ *n* = 931), a decreasing symptom group (‘case‐risk‐risk’ *n* = 400), an increasing‐symptom group (‘low‐risk‐case’, *n* = 394) and an increasing‐then‐decreasing symptom group (‘risk‐case‐risk’, *n* = 606).

For social‐emotional‐behavioural difficulties, the best fitting model was a four‐trajectory model (Figure 1B) labelled according to the SDQ total scale thresholds (Youth in Mind). This model identified a low‐stable difficulties group (‘low‐low‐low’, *n* = 4938), a high‐stable difficulties group (‘high‐veryhigh‐veryhigh’, *n* = 1734), a decreasing‐difficulties group (‘veryhigh‐average‐low’, *n* = 325), and an increasing‐difficulties group (‘low‐average‐veryhigh’, *n* = 700).

For wellbeing, the best fitting model was a four‐trajectory model (Figure 1C), labelled according to the WEMWBS scale thresholds (Tennant et al., 2007). This model identified an average wellbeing group (‘average‐average‐average’, *n* = 6175), an increasing wellbeing group (‘probable‐average‐average’, *n* = 139) and two decreasing wellbeing groups (‘possible‐possible‐probable’, *n* = 1210 and ‘average‐possible‐probable’, *n* = 200).

For anxiety, the best fitting model was a four‐trajectory model (Figure 1D), labelled according to general patterns using RCADS anxiety scale raw scores continuously (Chorpi et al., 2000). This model identified a low‐stable symptom group (‘low‐low‐low’*, *n* = 5514), high‐stable symptom group (‘high‐high‐high’*, *n* = 789), decreasing‐symptom group (‘high‐moderate‐moderate’, *n* = 354) and increasing‐symptom group (‘moderate‐high‐high’, *n* = 541). (*Note that the descriptors of ‘high’, ‘moderate’ and ‘low’ should be interpreted with caution given that raw scores were used for the RCADS rather than t‐scores; these labels do not necessarily equate to quantitative clinical severity).

While the above analyses describe trajectory memberships for each outcome separately, we also explored patterns in adolescent's trajectories across the four outcomes. Across all outcomes (*n* = 7188), 56.7% (*n* = 4080) of adolescents followed trajectories characterised by low‐stable or decreasing problems, and 43.2% (*n* = 3108) of adolescents followed at least one trajectory, across all outcomes, characterised by high‐stable or increasing problems.

### Predictors of trajectory membership

**FIGURE 2:**
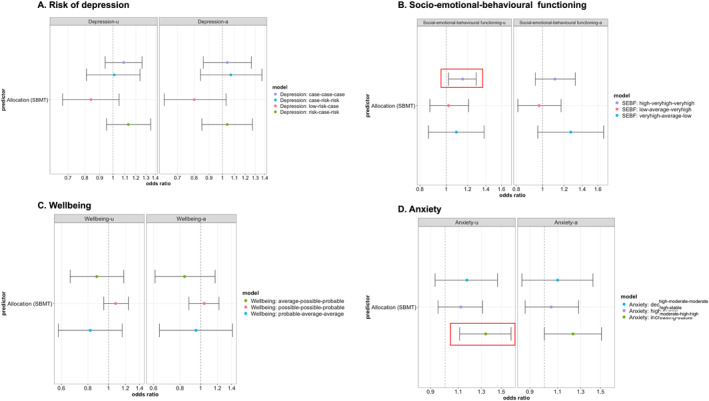
Unadjusted and adjusted models showing if allocation condition predicts trajectory membership. Unadjusted models only include allocation condition as a predictor whereas adjusted models are controlled for all other variables as predictors. Red boxes are showing significant effects in unadjusted models.

We examined whether allocation condition was associated to trajectory memberships using multinomial regressions analysis (Figure 2 and Supporting Information S1: Table S6–S9). The reference group was defined as the most common trajectory for each outcome (low‐stable trajectories for mental health scales and average‐stable trajectory for wellbeing). We found that for social‐emotional‐behavioural difficulties, individuals in the SBMT intervention condition compared to the control condition were more likely to follow a high‐stable (‘high‐veryhigh‐veryhigh’) trajectory compared to a low‐stable (‘low‐low‐low’) trajectory (OR = 1.15, CI = 1.02–1.29, *p* = 0.026). For anxiety, individuals in the SBMT intervention condition compared to the control condition were more likely to follow an increasing‐symptom (‘increasing‐stable’) trajectory compared to a low‐stable (‘low‐stable’) trajectory (OR = 1.34, CI = 1.11–1.61, *p* = 0.004). However, for both of these outcomes, associations were only significant in the unadjusted models and were no longer significant in the adjusted model (Figure 2). For risk of depression and wellbeing, intervention condition was not associated with any trajectory group in either unadjusted or adjusted models (all ps > 0.09).

We also conducted secondary direct comparisons between all decreasing and increasing symptom trajectory groups for each outcome (see Supporting Information S1). For risk of depression, we found one significant association in the unadjusted model such that young people allocated to the intervention were more likely to follow an increasing‐then‐decreasing symptom trajectory (‘risk‐case‐risk’) compared to an increasing‐symptom trajectory (‘low‐risk‐case’; OR = 1.37, CI = 1.06–1.77, *p* = 0.02), however this association became non‐significant in the adjusted model (Supporting Information S1: Table S10). We found no significant associations between intervention condition and trajectory group for any other decreasing/increasing trajectory comparisons (Supporting Information S1: Tables S11–S17), or interactions between different predictors and intervention condition.

Several other factors predicted young people's classification into different trajectories, but they did not vary according to intervention group (for all model results see Supporting Information S1). For both risk of depression and anxiety, young people in *all* other groups compared to the low‐stable reference group were more likely to have higher executive function difficulties, more likely to identify as female and more likely to be at risk of mental health problems at baseline (Supporting Information S1: Table S18 and S21). For risk of depression, individuals in the ‘risk‐case‐risk’ were more likely to go to school in urban locations and those in ‘case‐risk‐risk’ group more likely to experience a poorer school climate, both compared to the low‐stable reference group. Moreover, for anxiety, individuals in the ‘high‐moderate‐moderate’ and ‘moderate‐high‐high’ groups were more likely to be younger than individuals in the low‐stable reference group and individuals in the ‘moderate‐high‐high’ group were more likely to go to school in an urban area compared to individuals in the low‐stable reference group (Supporting Information S1: Table S21). For social‐emotional‐behavioural functioning, young people in *all* other groups compared to the low‐stable reference group were more likely to have higher executive function difficulties and more likely to be at risk of mental health problems at baseline, however there were other significant outcomes for individual trajectories compared to the low‐stable reference group including school climate, eFSM, gender and age (Supporting Information S1: Table S19 for all results). For wellbeing outcomes, individuals in the ‘possible‐possible‐probable’ and ‘probable‐average‐average’ groups were more likely to have higher executive function difficulties and more likely to be at risk of mental health problems at baseline, whilst individuals in the former were also more likely to be female than individuals in the low‐stable reference group (Supporting Information S1: Table S20).

## DISCUSSION

MYRIAD, a large 1‐year school‐based mindfulness intervention trial, found on average, null effects across all primary outcomes: risk for depression, social‐emotional‐behavioural functioning and wellbeing (Kuyken et al., 2022). However, these results tell us little, if anything, about the individual differences that predict symptom outcomes over time and in response to the intervention. Therefore, in this study, we explored whether different groups of participants responded differently to the intervention over time, and whether individual‐level and contextual‐level factors were associated with different symptoms trajectories. We identified different trajectories across all outcomes, suggesting considerable heterogeneity in this sample. In the unadjusted models, intervention allocation was associated with socio‐emotional‐behavioural functioning difficulties, anxiety and some risk of depression trajectories. However, after adjusting for multiple variables (gender, age, ethnicity, executive function difficulties, risk of mental health problems, school climate, school‐level deprivation, and urbanicity), we found that receiving the intervention was not associated with specific mental health trajectories from pre‐intervention to 12‐month follow‐up. Furthermore, several factors including greater executive function difficulties and risk of mental health problems predicted adolescents’ classification into different trajectories, but they did not vary according to intervention group. This indicates that the null average effects found in the MYRIAD trial were not concealing important trajectories followed by different subgroups of adolescents that were more or less likely to experience benefits (or harms) from this intervention over time.

There are several implications from these results. First, there is wide variation in young people's trajectories of mental health across a 1‐year period, which is consistent with prior studies examining mental health trajectories across different populations (Korhonen et al., 2014; Musliner et al., 2016; Schubert et al., 2017; Shore et al., 2018). Our growth mixture models showed that while most young people followed low‐stable problem trajectories over time in each outcome (69%–80%), a significant portion also followed high‐stable problem trajectories (11%–23%) and smaller groups followed increasing‐problem trajectories (2%–16%), decreasing‐problem trajectories (1%–5%) or a combination of both increasing and decreasing problems (8%). It is notable that most young people in our sample followed a stable trajectory of good mental health across the study period, while a minority fluctuated from experiencing low‐to‐high or high‐to‐low symptoms.

This raises the question on whether delivering mindfulness practices universally (i.e. at the same dose to everyone regardless of need) is a useful and cost‐effective approach. This aligns with other research indicating that targeted interventions, in which specific individuals at risk of developing mental health problems receive an intervention while others do not, might have larger effects and be more cost‐effective (Le et al., 2021). Perhaps universal interventions designed to prevent or reduce problems should not focus only on teaching therapeutic mental health concepts/strategies (such as mindfulness), but instead focus on promoting general positive health or social behaviours, which can act as protective factors to buffer and prevent any mental health problems. Another option in line with primary prevention might be to intervene earlier as most mental health problems arise by the age of 14 (Solmi et al., 2022). Other considerations such as how the intervention is designed (i.e. ideally by co‐production with key stakeholders and young people) and who is delivering the intervention (i.e. ideally by certified instructors rather than classroom teachers) might also make a difference to the success of any intervention (Cuijpers, 2022). Ultimately, understanding the mechanisms by which universal and targeted interventions work and the specific environments, individual characteristics and social determinants of mental health that triggers these mechanisms will allow us to determine what approach works best and for whom (Foulkes & Andrews, 2024; McGorry et al., 2024).

Lastly, our findings show that across intervention conditions, several individual‐level and school‐level factors (executive functioning difficulties, risk of mental health problems at baseline, gender, age, school climate, eFSM, and urbanicity) were associated with different trajectories and thus provide unique insights as to what factors were associated with an improvement or decline in young people's mental health over time. Some of these factors could be targeted prior to delivering a mindfulness intervention, or the intervention could be tailored based on characteristics of the sample to improve outcomes. For example, our results indicated that across *all* outcomes, when compared to the low stable groups, individuals in all other trajectory groups reported higher levels of executive function difficulties and greater risk of mental health problems at baseline. Furthermore, young people starting off in trajectories of high or very high risk for depression or socio‐emotional‐behavioural difficulties (regardless of whether the trajectory remained stable or decreased/increased) reported experiencing a more negative school climate than young people in low stable trajectories.

Thus, potentially the combination of having higher levels of mental health difficulties, poorer executive function and experiencing a negative school climate might contribute to young people feeling less inclined to engage in mindfulness activities in school, and later at home where they can continue to practice in daily life. As a result, these individuals might be less likely to experience a change in outcomes. Targeting factors such as school climate and teachers' engagement in procuring a positive and safe environment prior to an intervention, or modifying the intervention to be suitable for individuals with different pre‐existing levels of mental health problems or executive functioning difficulties, might improve their intervention outcomes and mental health trajectories. Other relevant factors not included in this study, which should be examined in future research, include the social context (Foulkes & Andrews, 2024), implementation factors (Montero‐Marin et al., 2023) or the age‐appropriateness of content delivered (Foulkes & Stapley, 2022).

Our findings should be understood in the context of some limitations. First, our trajectories were based on the entire sample of participants and analysed using an intent‐to‐treat approach. Different approaches such as instrumental variable analysis and analysis of treatment engagers should be considered in future studies to help disentangle whether the level of active engagement with an intervention, rather than allocation, can predict different trajectories of mental health outcomes. Indeed, student engagement (e.g. home practice) was considered ‘low’ in the main trial (Kuyken et al., 2022) with students practicing on average once during the intervention period (range 0–5) (Montero‐Marin et al., 2023). Thus, if we were to use a subsample of participants that completed all the mindfulness activities or had engagement levels greater than 80%, we might derive different trajectories or observe different interactions between predictors and intervention allocation. Second, it is important to note that trajectory classifications derived from LGMM are meant to reflect probabilistic group membership rather than deterministic assignment and should be interpreted accordingly. In other words, this means individuals are grouped based on likelihoods and there's always a change they could also fit into a different group. In terms of our predictor analysis, there may be other individual, school factors or implementation factors such as fidelity influencing trajectories that were not considered (Mansfield et al., 2023). Furthermore, we only examined baseline covariates and thus did not consider any changes to these factors that might have occurred throughout the study period. Lastly, the lack of extended longitudinal data prevents us from drawing conclusions about the longer‐term trajectories and any effects the intervention might have on these (e.g. sleeper effects).

## CONCLUSIONS

Investigating different symptom trajectories, especially as they relate to specific individual differences, is an essential step in better understanding how interventions work and for whom. This analysis of the MYRIAD trial highlights significant variability in the trajectories of risk for depression, social‐emotional‐behavioural functioning, and well‐being among adolescents over 1 year. While most participants maintained low‐stable symptom trajectories, up to one‐third of participants experienced increasing or high‐stable symptoms. After adjusting for multiple confounders, receiving the school‐based mindfulness intervention did not predict membership in any distinct trajectory of mental health, suggesting that universal interventions may not be sensitive enough to address the diverse needs of all students. Individual‐level factors, including executive functioning and risk of mental health problems along with school‐level factors such as school climate significantly influenced trajectory classifications, indicating that tailoring interventions to address these factors could enhance effectiveness.

## AUTHOR CONTRIBUTIONS

**Caroline Guzman Holst:** Conceptualization; formal analysis; investigation; methodology; project administration; visualization; writing—original draft; writing—review and editing. **Simona Skripkauskaite:** Conceptualization; methodology; writing—review and editing. **Jack L. Andrews:** Conceptualization; writing—review and editing. **Jesus Montero‐Marin:** Conceptualization; methodology; resources; writing—review and editing. **Verena Hinze:** Conceptualization; methodology; resources; writing—review and editing. **Tim Dalgleish:** Conceptualization; funding acquisition; writing—review and editing. **Willem Kuyken:** Conceptualization; funding acquisition; methodology; writing—review and editing. **Lucy Foulkes:** Conceptualization; investigation; methodology; project administration; supervision; writing—original draft; writing—review and editing.

## CONFLICT OF INTEREST STATEMENT

CGH, SS, JLA, JMM, VH, TD and LF report no conflicts of interest. WK is the Director of the Oxford Mindfulness Centre and receives royalties for several books on mindfulness. See Acknowledgements for full disclosures.

## ETHICAL CONSIDERATIONS

This study involves human participants and was approved by University of Oxford Central University Research Ethics Committee (R45358/RE001; 23/05/2016). Headteachers provided written consent for the participation of their schools in the study. Parents were provided with information about the trial and had the opportunity to opt their child out of the research study. Young people also provided informed consent prior to taking part in the baseline assessment of the study.

## Supporting information

Supporting Information S1

## Data Availability

No new data was generated for this study. Data from the MYRIAD study are available from Prof. Kuyken upon reasonable request (release of data is subject to an approved proposal and a signed data access agreement).
