# Supplementary material for: Adolescents' trajectories of mental health in the MYRIAD trial
Source: JCPP Adv. 2025 Oct 7;6(2):e70046. doi: 10.1002/jcv2.70046 (PMC7618703; doi:10.1002/jcv2.70046)
Supplement: Supplementary file 1 — Supporting Information S1 [file JCV2-6-e70046-s001.docx]

**Supplement**

**Supplement A. Methods**

*This supplement contains detailed information on design, participants and measures used in this study

Figure S1. Flowchart of study design

Table S2. Data availability from pre-intervention to 12-month follow-up for the analytic sample

Details of Measures used in this study

**Supplement B. Results 1**

*This supplement contains the results from LCGA/GMM model selection

Table S3: LCGA/GMM model selection for trajectories of risk for depression

Table S4: LCGA/GMM model selection for trajectories of SEBF

Table S5: LCGA/GMM model selection for trajectories of wellbeing

Table S6: LCGA/GMM model selection for trajectories of anxiety

**Supplement C. Results 2**

*This supplement contains the results from the primary and secondary analysis of multinomial/binomial regressions for each outcome

Table S7: Unadjusted and adjusted model for the effect of allocation condition on trajectory membership for risk of depression

Table S8: Unadjusted and adjusted model for the effect of allocation condition on trajectory membership for SEBF

Table S9: Unadjusted and adjusted model for the effect of allocation condition on trajectory membership for wellbeing

Table S10: Unadjusted and adjusted model for the effect of allocation condition on trajectory membership for anxiety

Table S11: Unadjusted and adjusted models for the effect of intervention allocation on the trajectory of RISK-CASE-RISK (risk of depression)

Table S12: Unadjusted and adjusted models for the effect of intervention allocation on the trajectory of LOW-RISK-CASE (risk of depression)

Table S13: Unadjusted and adjusted models for the effect of intervention allocation on the trajectory of RISK-CASE-RISK (risk of depression)

Table S14: Unadjusted and adjusted models for the effect of intervention allocation on the trajectory of LOW-AVERAGE-VERY HIGH (socio-emotional-behavioral functioning)

Table S15: Unadjusted and adjusted models for the effect of intervention allocation on the trajectory of PROBABLE-AVERAGE-AVERAGE (wellbeing)

Table S16: Unadjusted and adjusted models for the effect of intervention allocation on the trajectory of POSSIBLE-POSSIBLE-PROBABLE (wellbeing)

Table S17: Unadjusted and adjusted models for the effect of intervention allocation on the trajectory of PROBABLE-AVERAGE-AVERAGE (wellbeing)

Table S18: Unadjusted and adjusted models for the effect of intervention allocation on the trajectory of INCREASING STABLE (anxiety)

**Supplement D. Results 3**

*This supplement contains the results for all predictor analysis for each outcome using the low stable group as the reference

Table S19: Factors predicting trajectory membership for risk of depression

Table S20: Factors predicting trajectory membership for SEBF

Table S21: Factors predicting trajectory membership for wellbeing

Table S2: Factors predicting trajectory membership for anxiety

**Supplement A. Methods**

*This supplement contains detailed information on design, participants and measures used in this study

***Figure S1*.** Flowchart of study design


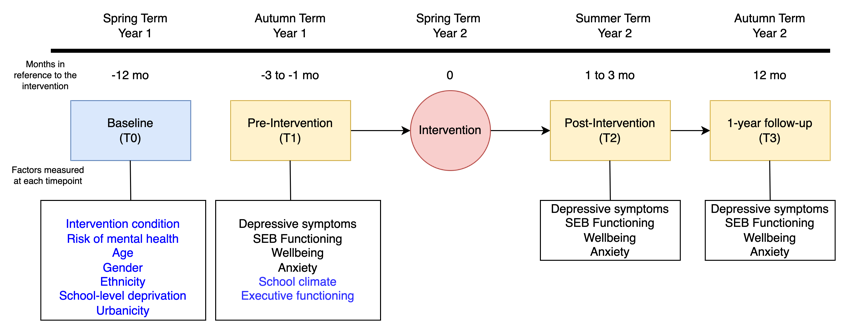


*Note*: the blue font indicates factors that were examined as predictors of trajectories, the black font indicates factors that were examined as outcomes

**Table S2**

*Data availability from pre-intervention to 12-month follow-up for the analytic sample*

|  | Pre-intervention | Post-intervention | 12-month follow-up | Total analytic sample |
| --- | --- | --- | --- | --- |
| Risk for depression (CES-D) | 8054 | 7561 (93.9%) | 7238 (89.9%) | 7712 (95.8%) |
| Socio-emotional-behavioral functioning (SDQ) | 8042 | 7542 (93.8%) | 7225 (89.8%) | 7697 (95.7%) |
| Well-being (WEMWBS) | 8058 | 7572 (94.0%) | 7244 (89.9%) | 7724 (95.9%) |
| Anxiety (RCADS) | 7585 | 7175 (94.6 %) | 6987 (92.1%) | 7198 (94.9%) |

*Note*: CESD-D=Center for Epidemiological Studies for Depression Scale; SDQ=strengths and difficulties questionnaire; WEMWBS=Warwick-Edinburgh Mental Well-being Scale; RCADS=Revised Child Anxiety and Depression Scale. This table shows the number of individuals who completed each scale at pre-intervention, post-intervention and 12-month follow-up. The total analytic sample consists of participants who completed a measure at pre-intervention and at least one other timepoint (post-intervention, 12-month follow-up, or both). The percentages indicating data availability since pre-intervention.

**Details of Measures used in this study**

***Primary and secondary outcomes***

**Depressive symptoms:** Participants completed the Center for Epidemiological Studies for Depression Scale (CES-D; Radloff, 1991). It consists of 20 items that measure how often over the past week they have experienced symptoms of depression, such as poor sleep and hopelessness. Response options range from 0 to 3 for each item (0 = Rarely or none of the time, 1 = Some or a little of the time, 2 = Moderately or much of the time, 3 = Most or almost all of the time). Scores range from 0 to 60, with high scores indicating greater depressive symptoms. The CES-D provides cut-off scores for low risk of depression (0-15), at risk of depression (16-27) and caseness (28-60) that aid in identifying individuals at risk for clinical depression, with good sensitivity and specificity and high internal consistency (Lewinsohn, Seeley, Roberts, & Allen, 1997; Radloff, 1991). These cutoffs will be used to interpret latent trajectories. In line with other publications from the MYRIAD trial, we refer to depressive symptoms in this paper as ‘risk for depression’.

**Mental well-being:** Participants completed the Warwick-Edinburgh Mental Well-being Scale (WEMWBS; Tennant et al., 2007) which is a widely used measure of positive mental health over the last two weeks. The scale consists of 14 items with five response categories (1 = None of the time; 2 = Rarely, 3 = Some of the time, 4 = Often, 5 = All of the time), summed to provide a single score. Scores range from 14 – 70, with high scores indicating greater mental well-being. The WEMWBS provides cut-off scores for probable mental health difficulties (0-40), possible mental health difficulties (41-44), average mental well-being (45-59) and high well-being (60-70); these terms will be used to interpret latent trajectories (Tennant et al., 2007; Warwick Medical School, 2021). The scale has good test-retest reliability and high internal consistency (Tennant et al., 2007).

**Social-emotional-behavioural difficulties:** Participants completed the self-reported youth version of the Strengths and Difficulties Questionnaire (SDQ; Goodman, 1997, 2001). This questionnaire is widely used to measure mental health difficulties experienced over the last six months. This study will use four subscales: emotional symptoms, conduct problems, hyperactivity/inattention, and peer relationship problems, which are summed to generate a total difficulties score. Each subscale consists of five items scored using a 3-point Likert scale (0 = not at all, 1= somewhat true, 2 = certainly true). Total scores may range from 0 to 40, with high scores indicating greater difficulties. Participants with missing information will have their scores pro-rated following SDQ guidance if they completed at least three items per subscale. SDQ scores will be used continuously and interpreted based on the new 4-categorisation band for the self-reported SDQ: low (0-14), moderate (15-17), high (18-19) and very high (20-40).

**Anxiety symptoms:** Participants completed the anxiety subscales from the Revised Child Anxiety and Depression Scale (RCADS; Chorpita et al., 2000), which is a widely used measure of anxiety over an undefined period. A total anxiety scale is derived from the sum of five anxiety subscales (37-items): social phobia (9 items), panic disorder (9 items), separation anxiety (7 items), generalized anxiety (6 items) and obsessive-compulsive disorder (6 items). Each subscale has four response categories (0 = Never, 1 = Sometimes, 2 = Often, 3 = Always) for a total score of 111, with higher scores indicating greater anxiety symptoms. Raw scores will be analysed continuously. The scale has good internal consistency and concurrent validity with other anxiety measures (Chorpita et al., 2005). We use the descriptors ‘high’, ‘moderate’ and ‘low’ to describe trajectories, however, as noted in the manuscript, this should be interpreted with caution given that raw scores were used for the RCADS rather than t-scores; these labels do not necessarily equate to quantitative clinical severity.

***Individual-level predictors***

**Demographic information:** Participants were asked about their age since last birthday at baseline (11-15 years), gender (male, female, other/prefer not to say), and ethnicity (White British, Asian, Black, mixed/other ethnic minorities). If any group has a percentage of less than 10% of the sample they will be grouped together for simplicity of analysis.

**Executive functioning difficulties:** Participants completed the self-reported Behaviour Rating Inventory of Executive Function (BRIEF-2; Gioia et al., 2000). This 55-item questionnaire assesses everyday behaviours associated with executive function. There are three main indexes: the Behaviour Regulation Index (BRI), the Emotion Regulation Index (ERI); and the Cognitive Regulation Index (CRI). We will use total raw scores continuously (range: 52 –156), with higher scores indicating greater self-reported difficulties. If the total score is significantly related to a specific trajectory group, we will then add individual index scores to the model. Note that, distinct from the other predictors, executive function was assessed at pre-intervention rather than at baseline, so pre-intervention data will be used as a predictor.

**Risk of mental health problems**: This variable was obtained from a latent profile analysis (LPA) conducted in Montero-Marin et al. (2022) which identified two subgroups of participants in the MYRIAD study (“at risk” and “low risk”) derived from nine baseline characteristics. These included student characteristics (age, gender, ethnicity, social-emotional-behavioural functioning, risk for depression, and well-being), the school’s broader context (school urbanity), school community (school deprivation), and school operational features (school social-emotional learning (SEL) ethos). The key difference between the two groups is high versus low symptoms on the three primary mental health measures. For more information on the LPA see Supplement C and Supplement E in Montero-Marin et al (2022).

***School-level and broader contextual predictors***

**School climate:** Students were asked to complete information about their school climate using the Alaska School Climate and Connectedness Survey (school climate). This measure includes three subscales: school leadership and involvement, respectful climate, and peer climate and caring adults. Response options vary by subscale but range from 1 to 5 for each item and are summed to produce a total score, with higher scores representing a better school climate. For the analysis, total scores will be averaged across students within a school to obtain a school-level measure of school climate. Note that, distinct from the other predictors, school climate was assessed at pre-intervention rather than at baseline, so pre-intervention data as a predictor will be used in this analysis. To reduce biases related to student’s own mental health influencing their perception of school climate this measure was aggregated at the school-level.

**School-level deprivation:** Information regarding school-level deprivation was obtained by linking publicly available governmental data regarding free school meals to the school’s postcode. We will use the percentage of students eligible for free school meals (eFSM) as an indicator of school-level deprivation. In line with the main trial, this variable will be used continuously.

**Urbanicity:** Information about the schools’ broader context was examined using a measure of urbanicity collected at baseline (T0) which reflected whether the school was in a rural area or urban area (coded as urban = 0, rural = 1). This information was obtained by linking publicly available government data or through email correspondence with schools. The governmental defined urban/rural classification was used to define the urbanicity or rurality of the areas the participating schools resided within.

**Supplement B. Results 1**

*This supplement contains the results from LCGA/GMM model selection

**Table S3.**

*LCGA/GMM model selection for trajectories of risk for depression*

| **Model** | **Class** | **AIC** | **BIC** | **Entropy Index** | **VLMR- LRT** | **Notes** |
| --- | --- | --- | --- | --- | --- | --- |
| LCGA - L | 1 | 170991.024 | 171011.875 | --- | --- |  |
| LCGA - L | 2 | 162141.055 | 162182.759 | 0.851 | 8532.502, p <.001 |  |
| LCGA - L | 3 | 159731.964 | 159794.519 | 0.822 | 2360.927, p <.001 |  |
| LCGA - L | 4 | 159031.108 | 159114.515 | 0.793 | 681.477, p <.001 |  |
| LCGA - L | 5 | 158359.543 | 158463.801 | 0.791 | 653.237, p = .012 |  |
| LCGA - L | 6 | 158028.178 | 158153.287 | 0.775 | 325.253, p = .020 |  |
| LCGA - L | 7 | 157829.729 | 157975.690 | 0.786 | 197.108, p = .074 |  |
| LCGA - Q | 1 | 170985.675 | 171013.477 | --- | --- |  |
| LCGA - Q | 2 | 162118.233 | 162173.837 | 0.852 | 8634.275, p <.001 |  |
| LCGA - Q | 3 | 159673.254 | 159756.660 | 0.823 | 2386.326, p<.001 |  |
| LCGA - Q | 4 | 158944.629 | 159055.837 | 0.790 | 716.609, p<.001 |  |
| LCGA - Q | 5 | 158219.920 | 158358.931 | 0.797 | 712.799, p = .005 |  |
| LCGA - Q | 6 | 157819.173 | 157985.986 | 0.813 | 397.640, p < .001 |  |
| LCGA - Q | 7 | 157513.417 | 157708.032 | 0.816 | 305.231, p = .002 |  |
| LCGA - Q | 8 | 157172.838 | 157395.255 | 0.775 | 339.107, p = .074 |  |
| GMM- L | 1 | 160545.680 | 160587.383 | ---- | ---- |  |
| GMM- L | 2 | 159361.088 | 159423.642 | 0.746 | 1147.845, p < .001 |  |
| GMM- L | 3 | 158453.122 | 158536.529 | 0.780 | 881.150, p < .001 |  |
| GMM- L | 4 | 157713.743 | 157818.001 | 0.817 | 718.616, p < .001 |  |
| GMM- L | 5 | 157448.720 | 157573.829 | 0.797 | 261.293, p = 0.002 |  |
| GMM- L | 6 | 157157.581 | 157303.543 | 0.805 | 286.470, p = 0.001 |  |
| GMM- L | 7 | 157009.361 | 157176.174 | 0.802 | 148.683, p = 0.03 |  |
| GMM- L | 8 | 156864.058 | 157051.722 | 0.798 | 145.871, p = 0.24 |  |
| GMM - Q | 1 | 160522.525 | 160571.179 | --- | ---- |  |
| GMM - Q | 2 | 158870.886 | 158947.342 | 0.789 | 1614.543, p < .001 |  |
| GMM - Q | 3 | 158274.667 | 158378.925 | 0.801 | 587.801, p = 0.048 |  |
| GMM - Q | 4 | 157507.993 | 157640.054 | 0.836 | 753.624, p < .001 |  |
| GMM - Q | 5 | 157094.294 | 157254.156 | 0.817 | 410.241, p < .001 |  |
| GMM - Q | 6 | 156754.599 | 156942.263 | 0.824 | 338.247, p = 0.09 |  |
| GMM - Q | **7** | 156479.559 | 156695.025 | 0.832 | 275.349, p = 0.08 |  |

*Note:* LCGA = latent class growth analysis model; GMM = growth mixture model; L = linear; Q = quadratic; AIC = Akaike information criterion; BIC = Bayesian information criterion; SSA BIC = sample size adjusted Bayesian information criterion; VLMR-LRT = Vuong-Lo-Mendell-Rubin Likelihood Ratio Test. Given the visual heterogeneity in classes, we extended the upper class limit from 6 to 8 to rule out exclusion criteria (e.g non-significant VLMR-LRT, increase in BIC or class size N < 100).

**Table S4.**

*LCGA/GMM model selection for trajectories of socio-emotional-behavioural difficulties*

| **Model** | **Class** | **AIC** | **BIC** | **Entropy**  **Index** | **VLMR LRT** | **Notes** |
| --- | --- | --- | --- | --- | --- | --- |
| LCGA - L | 1 | 146973.577 | 146994.422 | --- | --- |  |
| LCGA - L | 2 | 138508.062 | 138549.754 | 0.799 | 8167.284, p < .001 |  |
| LCGA - L | 3 | 135457.856 | 135520.393 | 0.794 | 2946.451, p < .001 |  |
| LCGA - L | 4 | 134379.972 | 134463.355 | 0.770 | 1044.960, p < .001 |  |
| LCGA - L | 5 | 134077.835 | 134182.064 | 0.724 | 297.071, p < .001 |  |
| LCGA - L | 6 | 133749.100 | 133874.174 | 0.715 | 322.715, p = .440 |  |
| LCGA - Q | 1 | 146944.466 | 146972.260 | --- | ---- |  |
| LCGA - Q | 2 | 138433.298 | 138488.887 | 0.800 | 8287.633, p <.001 |  |
| LCGA - Q | 3 | 135347.847 | 135431.230 | 0.796 | 3009.378, p <.001 |  |
| LCGA - Q | 4 | 134253.526 | 134364.704 | 0.772 | 1072.361, p <.001 |  |
| LCGA - Q | 5 | 133944.156 | 134083.128 | 0.727 | 308.745, p < .001 |  |
| LCGA - Q | 6 | 133581.310 | 133748.076 | 0.720 | 360.768, p = .014 |  |
| LCGA - Q | 7 | 133335.205 | 133529.765 | 0.737 | 247.199, p < .001 |  |
| LCGA - Q | 8 | 133090.458 | 133312.813 | 0.761 | 245.878, p = .003 | Class N < 100 |
| GMM - L | 1 | 134269.731 | 134311.422 | --- | --- |  |
| GMM - L | 2 | 133895.530 | 133958.067 | 0.781 | 366.547, p = .004 |  |
| GMM - L | 3 | 133579.713 | 133663.096 | 0.595 | 310.260, p < .001 |  |
| GMM - L | 4 | 133320.838 | 133425.067 | 0.657 | 255.363, p < .001 |  |
| GMM - L | 5 | 133106.848 | 133231.923 | 0.682 | 212.090, p < .001 | Class N < 100 |
| GMM- L | 6 | 133012.650 | 133158.570 | 0.685 | 96.601, p = .080 | Class N < 100 |
| GMM - Q | 1 | 134149.460 | 134198.100 | --- | --- |  |
| GMM - Q | 2 | 133586.320 | 133662.755 | 0.571 | 555.617, p < .001 |  |
| GMM - Q | 3 | 133289.977 | 133394.206 | 0.722 | 296.072, p = 0.154 | Class N < 100 |
| GMM - Q | 4 | 133038.874 | 133170.897 | 0.770 | 252.061, p = 0.41 | Class N < 100 |

*Note:* LCGA = latent class growth analysis model; GMM = growth mixture model; L = linear; Q = quadratic; AIC = Akaike information criterion; BIC = Bayesian information criterion; SSA BIC = sample size adjusted Bayesian information criterion; VLMR-LRT = Vuong-Lo-Mendell-Rubin Likelihood Ratio Test. The upper limit of six was not reached for GMM-Q models as class size was N < 100. We explored an upper limit of 8 for LCGA-Q to rule out exclusion criteria (e.g. non-significant VLMR-LRT or class size N < 100).

**Table S5.**

*LCGA/GMM model selection for trajectories of wellbeing*

| **Model** | **Class** | **AIC** | **BIC** | **Entropy**  **Index** | **VLMR LRT** | **Notes** |
| --- | --- | --- | --- | --- | --- | --- |
| LCGA - L | 1 | 162333.711 | 162354.568 | --- | --- |  |
| LCGA - L | 2 | 156595.215 | 156636.927 | 0.721 | 5538.278, p < .001 |  |
| LCGA - L | 3 | 154655.848 | 154718.417 | 0.729 | 1875.531, p <.001 |  |
| LCGA - L | 4 | 154021.072 | 154104.497 | 0.707 | 617.773, p <.001 |  |
| LCGA - L | 5 | 153795.261 | 153899.542 | 0.744 | 223.489, p = .100 | Class N < 100 |
| LCGA - L | 6 | 153566.441 | 153691.579 | 0.736 | 226.390, p < .097 |  |
| LCGA - Q | 1 | 162322.869 | 162350.677 | --- | --- |  |
| LCGA - Q | 2 | 156567.096 | 156622.712 | 0.722 | 5607.184, p < .001 |  |
| LCGA - Q | 3 | 154620.774 | 154704.199 | 0.729 | 1901.227, p <.001 |  |
| LCGA - Q | 4 | 153982.384 | 154093.617 | 0.708 | 628.829, p <.001 |  |
| LCGA - Q | 5 | 153673.030 | 153812.072 | 0.754 | 308.732, p < .001 | Class N < 100 |
| LCGA - Q | 6 | 153442.468 | 153609.318 | 0.743 | 232.081, p = .083 | Class N < 100 |
| GMM - L | 1 |  |  |  |  |  |
| GMM - L | 2 | 153543.419 | 153605.988 | 0.891 | 352.130, p < .001 |  |
| GMM - L | 3 | 153329.311 | 153412.736 | 0.875 | 212.206, p < .001 |  |
| GMM - L | 4 | 153205.824 | 153310.105 | 0.676 | 124.839, p = .013 | Entropy low |
| GMM - L | 5 | 153128.222 | 153253.359 | 0.667 | 80.601, p = .049 | Class N < 100 |
| GMM - Q | 1 |  |  |  |  |  |
| GMM - Q | 2 | 153442.410 | 153518.883 | 0.888 | 422.951, p < .01 |  |
| GMM - Q | 3 | 153057.047 | 153161.329 | 0.915 | 382.676, p < .001 | Class N < 100 |
| GMM - Q | 4 | 152794.644 | 152926.734 | 0.742 | 263.057, p = .036 | Class N < 100 |
| GMM - Q | 5 | 152643.228 | 152803.126 | 0.891 | 155.085, p = .217 | Class N < 100 |

*Note:* LCGA = latent class growth analysis model; GMM = growth mixture model; L = linear; Q = quadratic; AIC = Akaike information criterion; BIC = Bayesian information criterion; SSA BIC = sample size adjusted Bayesian information criterion; VLMR-LRT = Vuong-Lo-Mendell-Rubin Likelihood Ratio Test. Upper limit of six not reached for GMM-L and GMM-Q models as class size N < 100.

**Table S6.**

*LCGA/GMM model selection for trajectories of anxiety*

| **Model** | **Class** | **AIC** | **BIC** | **Entropy**  **Index** | **VLMR LRT** | **Notes** |
| --- | --- | --- | --- | --- | --- | --- |
| LCGA - L | 1 | 182241.439 | 182262.084 | --- | ---- |  |
| LCGA - L | 2 | 173763.661 | 173804.950 | 0.857 | 8176.892, p <.001 |  |
| LCGA - L | 3 | 171151.094 | 171213.028 | 0.826 | 2523.844, p < .001 |  |
| LCGA - L | 4 | 170251.254 | 170333.832 | 0.786 | 873.074, p <.001 |  |
| LCGA - L | 5 | 169732.687 | 169835.910 | 0.807 | 505.591, p = .223 |  |
| LCGA - L | 6 | 169444.746 | 169568.614 | 0.825 | 283.308, p = .064 | Class N < 100 |
| LCGA - Q | 1 | 182223.482 | 182251.009 | --- | --- |  |
| LCGA - Q | 2 | 173698.173 | 173753.226 | 0.857 | 8299.688, p < .001 |  |
| LCGA - Q | 3 | 171061.750 | 171144.329 | 0.827 | 2572.026, p < .001 |  |
| LCGA - Q | 4 | 170140.653 | 170250.758 | 0.788 | 903.660, p < .001 |  |
| LCGA - Q | 5 | 169603.704 | 169741.335 | 0.803 | 530.030, p = .178 |  |
| LCGA - Q | 6 | 169171.668 | 169336.825 | 0.817 | 427.989, p = .033 | Class N < 100 |
| GMM - L | 1 | 171404.894 | 171446.183 | --- | --- |  |
| GMM - L | 2 | 170378.307 | 170440.241 | 0.749 | 995.235, p < .001 |  |
| GMM - L | 3 | 169666.014 | 169748.592 | 0.791 | 692.310, p < .001 |  |
| GMM - L | 4 | 169170.797 | 169274.021 | 0.823 | 483.086, p = .018 |  |
| GMM - L | 5 | 168708.209 | 168832.077 | 0.832 | 451.638, p = .002 | Class N < 100 |
| GMM - L | 6 | 168546.958 | 168691.470 | 0.810 | 161.201, p = .004 | Class N < 100 |
| GMM - Q | 1 | 171327.664 | 171375.834 | --- | --- |  |
| GMM - Q | 2 | 170079.817 | 170155.515 | 0.763 | 1221.46, p < .001 |  |
| GMM - Q | 3 | 169445.060 | 169548.283 | 0.799 | 625.160, p < .001 |  |
| GMM - Q | 4 | 168886.664 | 169017.414 | 0.831 | 550.889, p < .001 |  |
| GMM - Q | 5 | 168342.146 | 168500.422 | 0.853 | 537.391, p < .01 | Class N < 100 |
| GMM - Q | 6 | 168024.972 | 168210.774 | 0.846 | 316.272, p = .556 | Class N < 100 |

*Note:* LCGA = latent class growth analysis model; GMM = growth mixture model; L = linear; Q = quadratic; AIC = Akaike information criterion; BIC = Bayesian information criterion; SSA BIC = sample size adjusted Bayesian information criterion; VLMR-LRT = Vuong-Lo-Mendell-Rubin Likelihood Ratio Test.

**Supplement C. Results 2**

**Table S7.**

*Unadjusted and adjusted model for the effect of allocation condition on trajectory membership for risk for depression*

|  | **Risk for depression – Unadjusted Model** | | | | | **Risk for depression – Adjusted Model** | | | | |
| --- | --- | --- | --- | --- | --- | --- | --- | --- | --- | --- |
| *Predictors* | *Odds Ratios* | *std. error* | *95% CI* | *Statistic* | *p* | *Odds Ratios* | *std. error* | *95% CI* | *Statistic* | *p* |
| allocation [I] × case-case-case | 1.09 | 0.08 | 0.94 – 1.26 | 1.17 | 0.274 | 1.04 | 0.10 | 0.86 – 1.26 | 0.44 | 0.752 |
| allocation [I] × case-risk-risk | 1.01 | 0.11 | 0.81 – 1.24 | 0.05 | 0.957 | 1.07 | 0.13 | 0.84 – 1.37 | 0.55 | 0.682 |
| allocation [I] × low-risk-case | 0.84 | 0.10 | 0.67 – 1.05 | -1.53 | 0.201 | 0.80 | 0.10 | 0.63 – 1.03 | -1.76 | 0.144 |
| allocation [I] × risk-case-risk | 1.13 | 0.10 | 0.95 – 1.35 | 1.37 | 0.227 | 1.04 | 0.11 | 0.85 – 1.27 | 0.37 | 0.763 |
| Observations | 38560 | | | | | 30855 | | | | |
| McFadden's R2 | 0.071 | | | | | 0.231 | | | | |

*Note*: CI = 95% confidence intervals

**Table S8.**

*Unadjusted and adjusted model for the effect of allocation condition on trajectory membership for socio-emotional-behavioral difficulties*

|  | **Socio-emotional-behavioral difficulties –**  **Unadjusted model** | | | | | **Socio-emotional-behavioral difficulties – Adjusted model** | | | | |
| --- | --- | --- | --- | --- | --- | --- | --- | --- | --- | --- |
| Predictors | Odds Ratios | std. Error | CI | Statistic | p | Odds Ratios | std. Error | CI | Statistic | p |
| allocation [I] × high-veryhigh-veryhigh | 1.15 | 0.07 | 1.02 – 1.29 | 2.38 | **0.026** | 1.11 | 0.10 | 0.94 – 1.32 | 1.27 | 0.293 |
| allocation [I] × low-average-veryhigh | 1.02 | 0.09 | 0.87 – 1.21 | 0.28 | 0.776 | 0.97 | 0.09 | 0.81 – 1.17 | -0.28 | 0.806 |
| allocation [I] × veryhigh-average-low | 1.09 | 0.13 | 0.86 – 1.38 | 0.71 | 0.573 | 1.27 | 0.18 | 0.96 – 1.68 | 1.70 | 0.140 |
| Observations | 30788 | | | | | 24684 | | | | |
| McFadden's R2 | 0.060 | | | | | 0.318 | | | | |

*Note*: CI = 95% confidence intervals

**Table S9.**

*Unadjusted and adjusted model for the effect of allocation condition on trajectory membership for wellbeing*

|  | **Wellbeing – Unadjusted model** | | | | | **Wellbeing – Adjusted model** | | | | |
| --- | --- | --- | --- | --- | --- | --- | --- | --- | --- | --- |
| Predictors | Odds Ratios | std. Error | CI | Statistic | p | Odds Ratios | std. Error | CI | Statistic | p |
| allocation [I] × ave-poss-prob | 0.88 | 0.13 | 0.66 – 1.18 | -0.85 | 0.397 | 0.84 | 0.14 | 0.61 – 1.17 | -1.03 | 0.563 |
| allocation [I] × poss-poss-prob | 1.08 | 0.07 | 0.95 – 1.24 | 1.17 | 0.319 | 1.04 | 0.09 | 0.88 – 1.22 | 0.47 | 0.772 |
| allocation [I] × prob-ave-ave | 0.82 | 0.15 | 0.58 – 1.16 | -1.11 | 0.319 | 0.95 | 0.19 | 0.64 – 1.41 | -0.24 | 0.900 |
| Observations | 30896 | | | | | 24696 | | | | |
| McFadden's R2 | 0.088 | | | | | 0.244 | | | | |

*Note*: CI = 95% confidence intervals

**Table S10.**

*Unadjusted and adjusted model for the effect of allocation condition on trajectory membership for anxiety*

|  | **Anxiety – Unadjusted model** | | | | | **Anxiety – Adjusted model** | | | | |
| --- | --- | --- | --- | --- | --- | --- | --- | --- | --- | --- |
| Predictors | Odds Ratios | std. Error | CI | Statistic | p | Odds Ratios | std. Error | CI | Statistic | p |
| allocation [I] × decreasing stable | 1.17 | 0.14 | 0.93 – 1.46 | 1.32 | 0.188 | 1.10 | 0.15 | 0.85 – 1.42 | 0.70 | 0.645 |
| allocation [I] × high-high-high | 1.12 | 0.09 | 0.95 – 1.31 | 1.37 | 0.188 | 1.05 | 0.10 | 0.87 – 1.28 | 0.54 | 0.686 |
| allocation [I] × increasing stable | 1.34 | 0.13 | 1.11 – 1.61 | 3.04 | **0.004** | 1.23 | 0.13 | 1.00 – 1.51 | 1.98 | 0.090 |
| Observations | 28792 | | | | | 24452 | | | | |
| McFadden's R2 | 0.077 | | | | | 0.268 | | | | |

| **Table S11.**  *Unadjusted and adjusted models for the effect of intervention allocation on the trajectory of RISK-CASE-RISK (risk for depression).* | | | | | | | | | | |
| --- | --- | --- | --- | --- | --- | --- | --- | --- | --- | --- |
|  | **Unadjusted model** | | | | | **Adjusted model** | | | | |
| *Predictors* | *Odds Ratios* | *std. Error* | *95%CI* | *Statistic* | *p* | *Odds Ratios* | *std. Error* | *95%CI* | *Statistic* | *p* |
| (Intercept) | 1.31 | 0.12 | 1.10 – 1.57 | 3.01 | **0.005** | 1.36 | 0.41 | 0.75 – 2.47 | 1.02 | 0.434 |
| Allocation [I] | 1.37 | 0.18 | 1.06 – 1.77 | 2.43 | **0.015** | 1.33 | 0.19 | 1.00 – 1.77 | 1.94 | 0.260 |
| Observations | 958 | | | | | 818 | | | | |
| R^2^ Tjur | 0.011 | | | | | 0.023 | | | | |

Note: The reference category is low-risk-case. CI = 95% confidence intervals

| **Table S12.**  *Unadjusted and adjusted models for the effect of intervention allocation on the trajectory of LOW-RISK-CASE (risk for depression)* | | | | | | | | | | |
| --- | --- | --- | --- | --- | --- | --- | --- | --- | --- | --- |
|  | **Unadjusted model** | | | | | **Adjusted model** | | | | |
| *Predictors* | *Odds Ratios* | *std. Error* | *95%CI* | *Statistic* | *p* | *Odds Ratios* | *std. Error* | *95%CI* | *Statistic* | *p* |
| (Intercept) | 1.08 | 0.11 | 0.89 – 1.31 | 0.74 | 0.459 | 0.95 | 0.32 | 0.49 – 1.85 | -0.15 | 0.979 |
| Allocation [I] | 0.83 | 0.12 | 0.63 – 1.10 | -1.29 | 0.397 | 0.71 | 0.12 | 0.51 – 0.99 | -2.02 | 0.146 |
| Observations | 794 | | | | | 648 | | | | |
| R^2^ Tjur | 0.002 | | | | | 0.115 | | | | |

Note: The reference category is case-risk-risk. CI = 95% confidence intervals

| **Table S13.**  *Unadjusted and adjusted models for the effect of intervention allocation on the trajectory of RISK-CASE-RISK (risk for depression)* | | | | | | | | | | |
| --- | --- | --- | --- | --- | --- | --- | --- | --- | --- | --- |
|  | **Unadjusted model** | | | | | **Adjusted model** | | | | |
| *Predictors* | *Odds Ratios* | *std. Error* | *95%CI* | *Statistic* | *p* | *Odds Ratios* | *std. Error* | *95%CI* | *Statistic* | *p* |
| (Intercept) | 1.41 | 0.13 | 1.18 – 1.70 | 3.73 | **<0.001** | 1.19 | 0.37 | 0.65 – 2.18 | 0.56 | 0.718 |
| Allocation [I] | 1.14 | 0.15 | 0.89 – 1.47 | 1.02 | 0.306 | 0.99 | 0.15 | 0.74 – 1.33 | -0.04 | 0.970 |
| Observations | 1006 | | | | | 810 | | | | |
| R^2^ Tjur | 0.001 | | | | | 0.065 | | | | |

Note: The reference category is case-risk-risk. CI = 95% confidence intervals.

| **Table S14**  *Unadjusted and adjusted models for the effect of intervention allocation on the trajectory of LOW-AVERAGE-VERY HIGH (socio-emotional-behavioral functioning)* | | | | | | | | | | |
| --- | --- | --- | --- | --- | --- | --- | --- | --- | --- | --- |
|  | **Unadjusted model** | | | | | **Adjusted model** | | | | |
| *Predictors* | *Odds Ratios* | *std. Error* | *95%CI* | *Statistic* | *p* | *Odds Ratios* | *std. Error* | *95%CI* | *Statistic* | *p* |
| (Intercept) | 2.26 | 0.22 | 1.87 – 2.75 | 8.39 | **<0.001** | 1.31 | 0.44 | 0.69 – 2.53 | 0.82 | 0.455 |
| Allocation [I] | 0.91 | 0.12 | 0.70 – 1.18 | -0.71 | 0.479 | 0.85 | 0.14 | 0.62 – 1.18 | -0.97 | 0.423 |
| Observations | 1025 | | | | | 826 | | | | |
| R^2^ Tjur | 0.000 | | | | | 0.142 | | | | |

Note: Reference category is very high-average-low. CI = 95% confidence intervals.

| **Table S15**  *Unadjusted and adjusted models for the effect of intervention allocation on the trajectory of PROBABLE-AVERAGE-AVERAGE (wellbeing)* | | | | | | | | | | |
| --- | --- | --- | --- | --- | --- | --- | --- | --- | --- | --- |
|  | **Unadjusted model** | | | | | **Adjusted model** | | | | |
| *Predictors* | *Odds Ratios* | *std. Error* | *95%CI* | *Statistic* | *p* | *Odds Ratios* | *std. Error* | *95%CI* | *Statistic* | *p* |
| (Intercept) | 0.76 | 0.11 | 0.56 – 1.01 | -1.85 | 0.128 | 0.72 | 0.40 | 0.24 – 2.15 | -0.58 | 0.902 |
| Allocation [I] | 0.83 | 0.18 | 0.54 – 1.28 | -0.84 | 0.402 | 1.06 | 0.29 | 0.62 – 1.81 | 0.22 | 0.902 |
| Observations | 339 | | | | | 268 | | | | |
| R^2^ Tjur | 0.002 | | | | | 0.099 | | | | |

Note: Reference category is average-possible-probable. CI = 95% confidence intervals

| **Table S16**  *Unadjusted and adjusted models for the effect of intervention allocation on the trajectory of POSSIBLE-POSSIBLE-PROBABLE (wellbeing)* | | | | | | | | | | |
| --- | --- | --- | --- | --- | --- | --- | --- | --- | --- | --- |
|  | **Unadjusted model** | | | | | **Adjusted model** | | | | |
| *Predictors* | *Odds Ratios* | *std. Error* | *95%CI* | *Statistic* | *p* | *Odds Ratios* | *std. Error* | *95%CI* | *Statistic* | *p* |
| (Intercept) | 5.63 | 0.60 | 4.59 – 6.98 | **16.16** | **<0.001** | 5.58 | 2.15 | 2.67 – 12.09 | 4.47 | **<0.001** |
| Allocation [I] | 1.15 | 0.18 | 0.85 – 1.56 | 0.93 | 0.350 | 1.22 | 0.23 | 0.85 – 1.76 | 1.08 | 0.402 |
| Observations | 1410 | | | | | 1160 | | | | |
| R^2^ Tjur | 0.001 | | | | | 0.136 | | | | |

Note: Reference category is average-possible-probable. CI = 95% confidence intervals

| **Table S17**  *Unadjusted and adjusted models for the effect of intervention allocation on the trajectory of PROBABLE-AVERAGE-AVERAGE (wellbeing)* | | | | | | | | | | |
| --- | --- | --- | --- | --- | --- | --- | --- | --- | --- | --- |
|  | **Unadjusted model** | | | | | **Adjusted model** | | | | |
| *Predictors* | *Odds Ratios* | *std. Error* | *95%CI* | *Statistic* | *p* | *Odds Ratios* | *std. Error* | *95%CI* | *Statistic* | *p* |
| (Intercept) | 0.13 | 0.02 | 0.11 – 0.17 | -16.64 | **<0.001** | 0.10 | 0.04 | 0.04 – 0.22 | -5.45 | **<0.001** |
| Allocation [I] | 0.72 | 0.13 | 0.50 – 1.02 | -1.82 | 0.069 | 0.91 | 0.19 | 0.60 – 1.37 | -0.47 | 0.639 |
| Observations | 1349 | | | | | 1108 | | | | |
| R^2^ Tjur | 0.002 | | | | | 0.038 | | | | |

Note: Reference category is possible-possible-probable. CI = 95% confidence intervals

| **Table S18**  *Unadjusted and adjusted models for the effect of intervention allocation on the trajectory of INCREASING STABLE (anxiety)* | | | | | | | | | | |
| --- | --- | --- | --- | --- | --- | --- | --- | --- | --- | --- |
|  | **Unadjusted model** | | | | | **Adjusted model** | | | | |
| *Predictors* | *Odds Ratios* | *std. Error* | *95%CI* | *Statistic* | *p* | *Odds Ratios* | *std. Error* | *95%CI* | *Statistic* | *p* |
| (Intercept) | 1.46 | 0.15 | 1.20 – 1.78 | 3.78 | **<0.001** | 1.63 | 0.57 | 0.83 – 3.23 | 1.40 | 0.377 |
| Allocation [I] | 1.09 | 0.15 | 0.83 – 1.42 | 0.61 | 0.543 | 1.08 | 0.18 | 0.78 – 1.50 | 0.45 | 0.652 |
| Observations | 895 | | | | | 763 | | | | |
| R^2^ Tjur | 0.000 | | | | | 0.209 | | | | |

Note: Reference category is decreasing stable. CI = 95% confidence intervals

**Supplement D. Results 3**

**Table S19.**

*Factors predicting trajectory membership for risk for depression*

| **Predictors** | **Odds Ratios** | **std. Error** | **CI** | **Statistic** | **p** |
| --- | --- | --- | --- | --- | --- |
| (Intercept) × case-case-case | 0.44 | 0.09 | 0.29 – 0.64 | -4.17 | **<0.001** |
| (Intercept) × case-risk-risk | 0.18 | 0.05 | 0.11 – 0.30 | -6.64 | **<0.001** |
| (Intercept) × low-risk-case | 0.16 | 0.04 | 0.10 – 0.27 | -7.05 | **<0.001** |
| (Intercept) × risk-case-risk | 0.23 | 0.05 | 0.15 – 0.35 | -6.75 | **<0.001** |
| allocation [I] × case-case-case | 1.04 | 0.10 | 0.86 – 1.26 | 0.44 | 0.752 |
| allocation [I] × case-risk-risk | 1.07 | 0.13 | 0.84 – 1.37 | 0.55 | 0.682 |
| allocation [I] × low-risk-case | 0.80 | 0.10 | 0.63 – 1.03 | -1.76 | 0.144 |
| allocation [I] × risk-case-risk | 1.04 | 0.11 | 0.85 – 1.27 | 0.37 | 0.763 |
| age × case-case-case | 0.92 | 0.07 | 0.79 – 1.07 | -1.02 | 0.470 |
| age × case-risk-risk | 0.93 | 0.09 | 0.77 – 1.13 | -0.71 | 0.599 |
| age × low-risk-case | 0.82 | 0.08 | 0.67 – 1.00 | -1.98 | 0.091 |
| age × risk-case-risk | 0.92 | 0.07 | 0.78 – 1.08 | -1.07 | 0.456 |
| ethnicity [W] × case-case-case | 0.90 | 0.10 | 0.72 – 1.13 | -0.88 | 0.509 |
| ethnicity [W] × case-risk-risk | 0.89 | 0.13 | 0.66 – 1.19 | -0.81 | 0.541 |
| ethnicity [W] × low-risk-case | 0.95 | 0.14 | 0.71 – 1.27 | -0.35 | 0.763 |
| ethnicity [W] × risk-case-risk | 1.02 | 0.12 | 0.80 – 1.29 | 0.16 | 0.877 |
| gender [M] × case-case-case | 0.22 | 0.03 | 0.18 – 0.28 | -13.19 | **<0.001** |
| gender [M] × case-risk-risk | 0.45 | 0.06 | 0.35 – 0.59 | -5.94 | **<0.001** |
| gender [M] × low-risk-case | 0.47 | 0.06 | 0.36 – 0.61 | -5.69 | **<0.001** |
| gender [M] × risk-case-risk | 0.37 | 0.04 | 0.30 – 0.46 | -8.82 | **<0.001** |
| efsm × case-case-case | 0.99 | 0.01 | 0.98 – 1.01 | -1.09 | 0.456 |
| efsm × case-risk-risk | 0.98 | 0.01 | 0.97 – 1.00 | -2.12 | 0.068 |
| efsm × low-risk-case | 1.01 | 0.01 | 0.99 – 1.02 | 0.96 | 0.484 |
| efsm × risk-case-risk | 0.99 | 0.01 | 0.98 – 1.01 | -0.98 | 0.484 |
| urbanicity [U] × case-case-case | 1.23 | 0.18 | 0.92 – 1.65 | 1.39 | 0.288 |
| urbanicity [U] × case-risk-risk | 1.13 | 0.21 | 0.78 – 1.63 | 0.64 | 0.637 |
| urbanicity [U] × low-risk-case | 1.07 | 0.20 | 0.75 – 1.54 | 0.39 | 0.763 |
| urbanicity [U] × risk-case-risk | 1.51 | 0.24 | 1.10 – 2.07 | 2.57 | **0.022** |
| risk profile [LR] × case-case-case | 0.20 | 0.02 | 0.16 – 0.24 | -16.03 | **<0.001** |
| risk profile [LR] × case-risk-risk | 0.36 | 0.05 | 0.28 – 0.47 | -7.75 | **<0.001** |
| risk profile [LR] × low-risk-case | 0.56 | 0.08 | 0.42 – 0.74 | -4.02 | **<0.001** |
| risk profile [LR] × risk-case-risk | 0.41 | 0.05 | 0.33 – 0.51 | -7.98 | **<0.001** |
| school climate × case-case-case | 0.48 | 0.16 | 0.26 – 0.91 | -2.26 | 0.051 |
| school climate × case-risk-risk | 0.33 | 0.14 | 0.14 – 0.73 | -2.70 | **0.016** |
| school climate × low-risk-case | 0.93 | 0.39 | 0.41 – 2.11 | -0.18 | 0.877 |
| school climate × risk-case-risk | 1.36 | 0.47 | 0.70 – 2.66 | 0.90 | 0.509 |
| EFD × case-case-case | 1.07 | 0.00 | 1.06 – 1.07 | 23.37 | **<0.001** |
| EFD × case-risk-risk | 1.05 | 0.00 | 1.05 – 1.06 | 15.16 | **<0.001** |
| EFD × low-risk-case | 1.02 | 0.00 | 1.02 – 1.03 | 6.31 | **<0.001** |
| EFD × risk-case-risk | 1.03 | 0.00 | 1.02 – 1.03 | 9.34 | **<0.001** |
| Observations 30855  McFadden's R^2.^ 0.231 | | | | | |

Note: EFD=executive functioning difficulties. None of the interactions with allocation are significant

**Table S20.**

*Factors predicting trajectory membership for socio-emotional-behavioral functioning*

| **Predictors** | **Odds Ratios** | **std. Error** | **CI** | **Statistic** | **p** |
| --- | --- | --- | --- | --- | --- |
| (Intercept) × high-veryhigh-veryhigh | 0.36 | 0.06 | 0.25 – 0.51 | -5.69 | **<0.001** |
| (Intercept) × low-average-veryhigh | 0.26 | 0.05 | 0.18 – 0.38 | -6.83 | **<0.001** |
| (Intercept) × veryhigh-average-low | 0.17 | 0.05 | 0.10 – 0.30 | -6.32 | **<0.001** |
| allocation [I] × high-veryhigh-veryhigh | 1.11 | 0.10 | 0.94 – 1.32 | 1.27 | 0.293 |
| allocation [I] × low-average-veryhigh | 0.97 | 0.09 | 0.81 – 1.17 | -0.28 | 0.806 |
| allocation [I] × veryhigh-average-low | 1.27 | 0.18 | 0.96 – 1.68 | 1.70 | 0.140 |
| age × high-veryhigh-veryhigh | 1.00 | 0.07 | 0.87 – 1.14 | -0.04 | 0.970 |
| age × low-average-veryhigh | 0.93 | 0.07 | 0.80 – 1.07 | -1.00 | 0.394 |
| age × veryhigh-average-low | 0.74 | 0.08 | 0.59 – 0.93 | -2.62 | **0.019** |
| ethnicity [W] × high-veryhigh-veryhigh | 1.21 | 0.13 | 0.99 – 1.49 | 1.84 | 0.110 |
| ethnicity [W] × low-average-veryhigh | 1.13 | 0.13 | 0.91 – 1.42 | 1.10 | 0.353 |
| ethnicity [W] × veryhigh-average-low | 0.89 | 0.15 | 0.64 – 1.24 | -0.68 | 0.565 |
| gender [M] × high-veryhigh-veryhigh | 0.56 | 0.05 | 0.47 – 0.66 | -6.54 | **<0.001** |
| gender [M] × low-average-veryhigh | 0.77 | 0.08 | 0.64 – 0.93 | -2.66 | **0.018** |
| gender [M] × veryhigh-average-low | 1.25 | 0.18 | 0.95 – 1.66 | 1.59 | 0.166 |
| efsm × high-veryhigh-veryhigh | 1.02 | 0.01 | 1.01 – 1.03 | 3.61 | **0.001** |
| efsm × low-average-veryhigh | 1.01 | 0.01 | 1.00 – 1.02 | 2.29 | **0.041** |
| efsm × veryhigh-average-low | 1.01 | 0.01 | 0.99 – 1.02 | 0.66 | 0.565 |
| urbanicity [U] × high-veryhigh-veryhigh | 1.32 | 0.17 | 1.02 – 1.71 | 2.14 | 0.058 |
| urbanicity [U] × low-average-veryhigh | 1.09 | 0.15 | 0.83 – 1.43 | 0.60 | 0.588 |
| Urbanicity [U] × veryhigh-average-low | 0.86 | 0.17 | 0.58 – 1.27 | -0.76 | 0.536 |
| risk profile [LR] × high-veryhigh-veryhigh | 0.19 | 0.02 | 0.16 – 0.23 | -18.43 | **<0.001** |
| risk profile [LR] × low-average-veryhigh | 0.42 | 0.05 | 0.34 – 0.52 | -7.92 | **<0.001** |
| risk profile [LR] × veryhigh-average-low | 0.19 | 0.03 | 0.14 – 0.26 | -11.28 | **<0.001** |
| school climate × high-veryhigh-veryhigh | 0.50 | 0.14 | 0.28 – 0.87 | -2.44 | **0.029** |
| school climate × low-average-veryhigh | 0.68 | 0.22 | 0.36 – 1.27 | -1.22 | 0.306 |
| school climate × veryhigh-average-low | 0.25 | 0.12 | 0.10 – 0.63 | -2.94 | **0.008** |
| EFD × high-veryhigh-veryhigh | 1.10 | 0.00 | 1.09 – 1.10 | 31.91 | **<0.001** |
| EFD × low-ave-veryhigh | 1.03 | 0.00 | 1.03 – 1.04 | 10.77 | **<0.001** |
| EFD × veryhigh-ave-low | 1.07 | 0.00 | 1.06 – 1.08 | 16.12 | **<0.001** |
| Observations | 24684 | | | | |
| McFadden's R2 | 0.318 | | | | |

Note: EFD=executive functioning difficulties. None of the interactions with allocation are significant

**Table S21.**

*Factors predicting trajectory membership for wellbeing*

| **Predictors** | **Odds Ratios** | **std. Error** | **CI** | **Statistic** | **p** |
| --- | --- | --- | --- | --- | --- |
| (Intercept) × ave-pos-prob | 0.05 | 0.02 | 0.03 – 0.10 | -8.53 | **<0.001** |
| (Intercept) × poss-poss-prob | 0.28 | 0.05 | 0.20 – 0.39 | -7.32 | **<0.001** |
| (Intercept) × prob-ave-ave | 0.03 | 0.01 | 0.01 – 0.07 | -8.41 | **<0.001** |
| allocation [I] × ave-pos-prob | 0.84 | 0.14 | 0.61 – 1.17 | -1.03 | 0.563 |
| allocation [I] × poss-poss-prob | 1.04 | 0.09 | 0.88 – 1.22 | 0.47 | 0.772 |
| allocation [I] × prob-ave-ave | 0.95 | 0.19 | 0.64 – 1.41 | -0.24 | 0.900 |
| age × ave-pos-prob | 0.80 | 0.11 | 0.62 – 1.05 | -1.60 | 0.272 |
| age × poss-poss-prob | 1.07 | 0.07 | 0.94 – 1.21 | 1.00 | 0.563 |
| age × prob-ave-ave | 1.03 | 0.16 | 0.76 – 1.41 | 0.20 | 0.900 |
| ethnicity [W] × ave-pos-prob | 0.90 | 0.17 | 0.62 – 1.32 | -0.54 | 0.772 |
| ethnicity [W] × poss-poss-prob | 1.09 | 0.11 | 0.89 – 1.33 | 0.85 | 0.612 |
| ethnicity [W] × prob-ave-ave | 0.77 | 0.18 | 0.49 – 1.21 | -1.15 | 0.538 |
| gender [M] × ave-pos-prob | 0.87 | 0.15 | 0.62 – 1.21 | -0.83 | 0.612 |
| gender [M] × poss-poss-prob | 0.36 | 0.03 | 0.30 – 0.43 | -10.86 | **<0.001** |
| gender [M] × prob-ave-ave | 1.02 | 0.21 | 0.69 – 1.52 | 0.11 | 0.944 |
| efsm × ave-pos-prob | 1.01 | 0.01 | 0.99 – 1.03 | 1.03 | 0.563 |
| efsm × poss-poss-prob | 1.00 | 0.00 | 0.99 – 1.01 | 0.52 | 0.772 |
| efsm × prob-ave-ave | 1.01 | 0.01 | 0.99 – 1.03 | 0.78 | 0.626 |
| urbanicity [U] × ave-pos-prob | 1.01 | 0.26 | 0.62 – 1.66 | 0.05 | 0.956 |
| urbanicity [U] × poss-poss-prob | 1.34 | 0.17 | 1.04 – 1.73 | 2.24 | 0.085 |
| urbanicity [U] × prob-ave-ave | 1.07 | 0.33 | 0.59 – 1.94 | 0.21 | 0.900 |
| risk profile [LR] × ave-pos-prob | 0.65 | 0.13 | 0.44 – 0.96 | -2.15 | 0.094 |
| risk profile [LR] × poss-poss-prob | 0.27 | 0.02 | 0.23 – 0.32 | -15.16 | **<0.001** |
| risk profile [LR] × prob-ave-ave | 0.45 | 0.10 | 0.29 – 0.68 | -3.74 | **0.001** |
| school climate × ave-pos-prob | 0.45 | 0.25 | 0.15 – 1.34 | -1.43 | 0.350 |
| school climate × poss-poss-prob | 0.58 | 0.16 | 0.34 – 1.00 | -1.96 | 0.137 |
| school climate × prob-ave-ave | 0.73 | 0.49 | 0.20 – 2.71 | -0.46 | 0.772 |
| executive dysfuntion × ave-pos-prob | 1.00 | 0.00 | 0.99 – 1.01 | 0.83 | 0.612 |
| executive dysfuntion × poss-poss-prob | 1.04 | 0.00 | 1.04 – 1.05 | 18.91 | **<0.001** |
| executive dysfuntion × prob-ave-ave | 1.04 | 0.01 | 1.02 – 1.05 | 6.72 | **<0.001** |
| Observations | 24696 | | | | |
| McFadden's R^2^ | 0.244 | | | | |

Note: EFD=executive functioning difficulties. None of the interactions with allocation are significant

**Table S22.**

*Factors predicting trajectory membership for anxiety*

| **Predictors** | **Odds Ratios** | **std. Error** | **CI** | **Statistic** | **p** |
| --- | --- | --- | --- | --- | --- |
| (Intercept) × decreasing-stable | 0.09 | 0.02 | 0.05 – 0.15 | -8.92 | **<0.001** |
| (Intercept) × high-stable | 0.20 | 0.04 | 0.14 – 0.31 | -7.72 | **<0.001** |
| (Intercept) × increasing-stable | 0.13 | 0.03 | 0.09 – 0.21 | -8.83 | **<0.001** |
| allocation [I] × decreasing-stable | 1.10 | 0.15 | 0.85 – 1.42 | 0.70 | 0.645 |
| allocation [I] × high-stable | 1.05 | 0.10 | 0.87 – 1.28 | 0.54 | 0.686 |
| allocation [I] × increasing-stable | 1.23 | 0.13 | 1.00 – 1.51 | 1.98 | 0.090 |
| age × decreasing-stable | 0.79 | 0.08 | 0.64 – 0.97 | -2.25 | **0.049** |
| age × high-stable | 1.03 | 0.08 | 0.88 – 1.20 | 0.34 | 0.771 |
| age × increasing-stable | 0.71 | 0.06 | 0.60 – 0.84 | -4.01 | **<0.001** |
| ethnicity [W] × decreasing-stable | 0.90 | 0.14 | 0.66 – 1.22 | -0.68 | 0.645 |
| ethnicity [W] × high-stable | 1.07 | 0.13 | 0.84 – 1.35 | 0.54 | 0.686 |
| ethnicity [W] × increasing-stable | 1.14 | 0.14 | 0.89 – 1.46 | 1.06 | 0.483 |
| gender [M] × decreasing-stable | 0.45 | 0.06 | 0.34 – 0.59 | -5.67 | **<0.001** |
| gender [M] × high-stable | 0.14 | 0.02 | 0.11 – 0.19 | -14.84 | **<0.001** |
| gender [M] × increasing-stable | 0.37 | 0.04 | 0.30 – 0.47 | -8.41 | **<0.001** |
| efsm × decreasing-stable | 1.00 | 0.01 | 0.98 – 1.01 | -0.53 | 0.686 |
| efsm × high-stable | 1.00 | 0.01 | 0.99 – 1.01 | -0.50 | 0.686 |
| efsm × increasing-stable | 1.01 | 0.01 | 1.00 – 1.02 | 1.23 | 0.384 |
| urbanicity [U] × decreasing-stable | 1.06 | 0.21 | 0.72 – 1.56 | 0.30 | 0.771 |
| urbanicity [U] × high-stable | 1.04 | 0.16 | 0.78 – 1.40 | 0.29 | 0.771 |
| urbanicity [U] × increasing-stable | 1.60 | 0.27 | 1.15 – 2.22 | 2.77 | **0.012** |
| risk profile [LR] × decreasing-stable | 0.47 | 0.07 | 0.36 – 0.61 | -5.46 | **<0.001** |
| risk profile [LR] × high-stable | 0.40 | 0.04 | 0.33 – 0.49 | -8.81 | **<0.001** |
| risk profile [LR] × increasing-stable | 0.50 | 0.06 | 0.40 – 0.63 | -5.89 | **<0.001** |
| school climate × decreasing-stable | 0.72 | 0.33 | 0.30 – 1.75 | -0.72 | 0.645 |
| school climate × high-stable | 0.71 | 0.24 | 0.37 – 1.38 | -1.00 | 0.486 |
| school climate × increasing-stable | 1.42 | 0.51 | 0.71 – 2.85 | 0.99 | 0.486 |
| EFD × decreasing-stable | 1.07 | 0.00 | 1.06 – 1.08 | 18.16 | **<0.001** |
| EFD × high-stable | 1.07 | 0.00 | 1.07 – 1.08 | 23.46 | **<0.001** |
| EFD × increasing-stable | 1.02 | 0.00 | 1.01 – 1.02 | 5.22 | **<0.001** |
| Observations | 24452 | | | | |
| McFadden's R^2^ | 0.268 | | | | |

Note: EFD=executive functioning difficulties. None of the interactions with intervention allocation are significant
